# On a path to becoming more self-regulated: Reflective journals’ impact on Chinese English as a foreign language students’ self-regulated writing strategy use

**DOI:** 10.3389/fpsyg.2022.1042031

**Published:** 2022-11-16

**Authors:** Yining Zhang, Mingzhu Li, YuanTzu Chieh, Shuyuan Han

**Affiliations:** Department of Foreign Languages and Literatures, Tsinghua University, Beijing, China

**Keywords:** self-regulated learning, self-regulated strategy use, L2 writing, reflective journals, Chinese EFL learning

## Abstract

A number of studies have confirmed the positive effect of writing reflective journals on L2 learning. However, the relationship between writing reflective journals and the use of self-regulated writing strategies remains unclear. To redress this knowledge gap, we assigned 38 Chinese English as a foreign language (EFL) students three journal-writing tasks in which they reflected on their writing processes and explored (1) the types of self-regulated writing strategies and changes to those strategies that the students’ reflective journals documented; (2) how students with varied writing-proficiency levels differed in their use of self-regulated writing strategies; and (3) the effects of reflective-journal writing on students’ self-perceived use of self-regulated writing strategies in particular, and on their L2 writing in general. Among the 19 kinds of strategies identified in 112 reflective-journal entries, only five (i.e., *handling feedback*, *resource management*, *text processing*, *emotion regulation*, and *idea planning*) were demonstrated relatively frequently. The use of seven strategies (i.e., *self-monitoring and evaluation*, *idea planning, perspective change*, *emotional control*, *effort regulation*, *peer learning*, and *resource management*) exhibited significant increases over time, especially during the second-half of the focal semester. In addition, our journal data highlighted individual variation in proficiency levels: with high-proficiency students significantly more likely than others to apply *idea planning, feedback handling*, and *resource management* and low-proficiency ones significantly more likely than others to engage in *goal-setting*. The qualitative results suggest that the practice of journaling raised students’ awareness and may have contributed to an increase in their use of self-regulated writing strategies. In particular, the findings reveal how students internalized and reconstructed the various SRL processes taking place *via* writing reflective journals. For L2 educators using or considering using reflective journals, these findings contain fresh insights that could help them not only to increase their students’ SRL levels, but also to provide more individualized SRL guidance.

## Introduction

Writing is arguably the most essential, yet the most challenging, second-language (L2) learning skill (Zhang and Guo, 2012). This is not only due to it requiring sufficient L2 language knowledge during the writing process (Manchón, 2011), but also because it is a highly structured process intertwined with individual and environmental factors such as motivation, working memory, cognitive and metacognitive processing, and the task environment (Flower and Hayes, 1981; Hayes, 1996; Winne and Hadwin, 1998). According to Zimmerman and Risemberg (1997), becoming an adept writer depends on high level use of self-regulated learning (SRL) strategies as writing is a goal-driven, self-initiated, and self-maintained activity.

Though the components of different scholars’ SRL models vary (e.g., Winne and Hadwin, 1998; Pintrich, 2000; Zimmerman, 2000), self-reflection is always a crucial phase of SRL development (Schunk and Ertmer, 2000). During this phase, learners not only ascribe reasons to their successes and failures (Zimmerman, 2000) but also make cognitive judgments, express affective reactions, engage in choice behavior, and evaluate tasks (Pintrich, 2004). To maximize the benefits of self-reflection, many L2 educators require their students to write reflective journals (e.g., Takeuchi, 2019; Sudirman et al., 2021). Although a number of studies have confirmed the effectiveness of writing reflective journals in L2 learning (e.g., Chang and Lin, 2014; Chang et al., 2015; Rassaei, 2015; Wu and Lin, 2015; Hussein, 2018; Baek, 2019; Hussein et al., 2020; Farahian et al., 2021; Lv et al., 2021; Sudirman et al., 2021), its relationship with self-regulated writing strategy use remains unclear.

A second gap in the existing literature is that few studies have explored how students writing reflective journals demonstrate and develop their awareness of different types of SRL-strategy use, or how individual learner differences influence self-regulated writing processes (Farahian et al., 2021). With regard to the latter, L2 proficiency level is an important individual-difference factor, yet findings about its influence are relatively limited (Yabukoshi, 2020). More studies of how students with varied writing-proficiency levels reflect on their writing processes *via* reflective journals are therefore warranted.

Accordingly, the present work assigned Chinese students of English as a foreign language (EFL) three journal-writing tasks, each consisting of reflection on their writing processes, and examined the resulting data for (1) the types of self-regulated writing strategies they used and changes to those strategies over time; (2) how students with varied writing-proficiency levels differed in their use of self-regulated writing strategies; and (3) the effects of reflective-journal writing on students’ self-perceived self-regulated writing strategy use in particular and on their self-perceived L2 writing in general. It is hoped that its findings will equip L2 educators who are considering using reflective journals with fresh insights that could help them increase their students’ SRL levels and provide more individualized SRL guidance.

## Literature review

### The use of self-regulated learning strategies in L2 writing

SRL in first-language (L1) writing has been studied widely (e.g., Zimmerman and Risemberg, 1997; Zimmerman and Kitsantas, 2002), but the equivalent body of work on SRL in L2 writing started relatively late and remains small (Bai and Guo, 2018). This is surprising, given that writing is an inherently complex activity that requires learners to exhibit strong cognitive as well as linguistic abilities (Hayes, 1996; Kellogg, 1996), and that L2 writers generally strive to obtain language knowledge during the writing process (Manchón, 2011). L2 writing is also time-consuming, requiring learners’ motivation and regulation (Kormos, 2012). Therefore, in theory, SRL could play a key role in determining how students initiate, sustain, regulate, and monitor their L2 writing processes.

Unlike classifications of L2 writing strategy from a cognitive perspective that classified writing strategies in terms of three cognitive phases (i.e., pre-writing, composing, and revising; e.g., Cumming, 1989; Wenden, 1991), some studies have sought to establish which strategies or other factors are key to L2 writing from a socio-cognitive angle (e.g., Kormos, 2012; Csizér and Tankó, 2017; Sun and Wang, 2020). This line of research integrated social and motivational aspect into writing strategy use by highlighting the important role of SRL in writing (e.g., Cumming et al., 2002). Such research was thus regarded as more comprehensive as it acknowledged “the multidimensional nature of the writing process” (Teng and Zhang, 2016a, p. 5). For example, following the framework created by Zimmerman and Risemberg (1997), Sun and Wang (2020) used a questionnaire covering three dimensions, i.e., environmental, behavioral, and personal factors, to measure English writers’ SRL strategies. In another study, Teng and Zhang (2016a) validated a questionnaire designed to measure SRL strategy use in EFL writing. They regarded self-regulation as a higher-order construct over nine lower-level writing strategies grouped according to whether they involved cognitive, metacognitive, social-behavioral or motivational regulation. In general, to achieve sustainable L2 writing, a person must actively employ four distinct types of self-regulated writing strategies: cognitive, metacognitive, behavioral, and motivational (e.g., Teng and Zhang, 2016a, 2018; Csizér and Tankó, 2017; Hu and Gao, 2018). Learners use cognitive strategies to learn to accomplish tasks using cognitive abilities such as knowledge processing, constructing, rehearsing, and transforming (Zimmerman, 2000); use metacognitive strategies to plan, monitor, control, and evaluate the entire learning process (Pintrich, 2000; Zimmerman, 2000); use behavioral strategies to select or create an adaptive learning environment (Pintrich, 2000); and finally, use motivational strategies to actively manage their motivation or motivational processing during learning (Zimmerman and Schunk, 2008).

In general, the quantity of using self-regulated writing strategies by L2 writers is moderate (e.g., Abadikhah et al., 2018; Bai et al., 2022). This also holds true of its subcomponents including cognitive strategies (Sethuraman and Radhakrishnan, 2020), planning (Bai and Guo, 2018, 2021; Bai et al., 2020), self-monitoring (Guo et al., 2021), help-seeking, and motivational regulation (Mbato and Cendra, 2019). On the other hand, some studies have reported that students did not fully adopt self-regulated writing strategies (e.g., Mallahi, 2020; Akhmedjanova and Moeyaert, 2022), with three strategies being especially poorly represented. They were goal-setting (Abadikhah et al., 2018; Sun and Wang, 2020), reviewing of records (Sun and Wang, 2020), and self-consequence (Abadikhah et al., 2018). Meanwhile, Xu (2021) found that Chinese university EFL students adopted fewer social-behavior strategies than other self-regulated writing strategies.

Studies of the associations between L2 learners’ writing proficiency and various aspects of self-regulated writing strategies have, in general, reported them to be positive (e.g., Teng and Zhang, 2016a; Hu and Gao, 2018; Bai et al., 2020; Jackson and Park, 2020; Sun and Wang, 2020; Bai and Guo, 2021; Guo and Bai, 2022; Shen and Bai, 2022). In particular, such findings have involved the strategies of noticing (Hu and Gao, 2018), selecting (Hu and Gao, 2018), text processing (Alanazi, 2020), memorizing (Hu and Gao, 2018; Alanazi, 2020), planning (Chien, 2012; Bai et al., 2020, 2022; Guo and Bai, 2022), text-generation (Chien, 2012; Bai et al., 2020, 2022), self-monitoring/evaluation (Hu and Gao, 2018; Bai et al., 2020, 2022; Sun and Wang, 2020; Guo and Bai, 2022), reviewing of records (Sun and Wang, 2020), seeking opportunities (Sun and Wang, 2020), revising (Chien, 2012; Bai et al., 2020; Guo and Bai, 2022), and motivational regulation (Teng and Zhang, 2018; Teng et al., 2020; Shen and Bai, 2022). However, some other studies have reported non-significant correlations between writing proficiency and the use of SRL strategies (e.g., Mallahi, 2020). For example, Csizér and Tankó (2017) found that even students with relatively high language proficiency might not be aware of the importance of SRL strategy use. Such results imply that levels of using self-regulated writing strategies and the relationship between strategy use and writing proficiency are both subject to marked individual and environmental variations (see also Inan-Karagul and Seker, 2021).

Regarding change over time in self-regulated writing strategies, SRL theory itself emphasizes the central role of time and ordered SRL sequencing during the writing process (Zimmerman, 2008; Zimmerman and Schunk, 2011), and that students’ journey to adopting such strategies may be quite lengthy (Akhmedjanova and Moeyaert, 2022). Therefore, it is important to trace developmental patterns in students’ use of them. Some studies have reported fluctuating use of self-regulated writing strategies over time: with Jackson and Park (2020), for example, capturing three participants’ dynamic views of SRL at nine time-points over a semester. In general, their results suggested that these individuals’ SRL did not increase much over the semester, and that the nature of its fluctuations varied from person to person. Similarly, Wilby (2020) reported that although students’ writing motivation and self-efficacy increased significantly over a semester, their metacognitive self-regulation skills (i.e., planning, monitoring, self-control, and self-reflection) did not exhibit significant change. Wilby speculated that this could have been due to their lack of opportunities to reflect on their work – a process that, according to Pintrich (2000), plays a key role in SRL. This further highlights the need to build reflection-related activity into L2 writing instruction. In a more recent study, Saqr et al. (2021) showed how SRL behavior deteriorated over time in an academic-writing class. Specifically, both writing arguments and reflecting were prominent during the students’ completion of their first assignment, but their incidence dropped during the second and the third assignments.

However, some other studies have suggested that self-regulated writing strategy use can improve over time. For example, Han and Hiver (2018) identified three profiles of learners based on their SRL, self-efficacy, and anxiety about English writing. The authors found that, throughout the focal semester, the students who fit each of these profiles either developed or consolidated their SRL. The same authors suggested that such improvement may have involved the instruction the students received, which scaffolded their attention to task requirements, use of linguistic resources, and choices as writers. In the same year, Sasaki et al. (2018) reported the results of their three-and-a-half-year observation of how Japanese EFL learners used three writing strategies: i.e., global planning, local planning, and L1-to-L2 translation. Their participants tended to use both global planning and local planning strategies more frequently as time went on, and their L2 writing proficiency also increased. The same authors also pointed out that their participants’ developmental pattern was not linear; that between-subjects variation overshadowed its within-subjects counterpart; and that this overshadowing became more marked with the passage of time. In sum, the change of self-regulated writing strategy use over time appears nearly impossible to generalize. Instead, it should be examined on a case-by-case basis, especially when considering individual difference and the effects of particular pedagogical practices, such as reflection.

### The use of reflective journals in L2 learning

Reflective journals are “written documents that students create as they think about various concepts, events, or interactions over a period of time for the purposes of gaining insights into self-awareness and learning” (Thorpe, 2004, p. 328). From a sociocultural perspective, keeping a reflective journal is a dialogic activity that triggers an interaction between a learner and him- or her-self (Rassaei, 2015). At the core of reflective-journal writing is one’s ability to reflect on one’s own learning process and progress and thus obtain new knowledge that can guide future learning actions (Moon, 2006; Chang and Lin, 2014; Rassaei, 2015; Hussein, 2018; Baek, 2019; Hussein et al., 2020; Sudirman et al., 2021). In this sense, reflective-journal writing would appear to be closely linked to the final self-reflection phase of Zimmerman’s (2000) cyclical modal of SRL.

Various advantages of reflective journals in L2 contexts have been documented, including their potential use as indices of improvement in writing effectiveness (Wu and Lin, 2015; Hussein et al., 2020; Lv et al., 2021; Sudirman et al., 2021), reading effectiveness (Chang and Lin, 2014), recast effectiveness (Rassaei, 2015), and a growth mindset (Hussein, 2018). Some studies have reported that the use of reflective writing can promote students’ SRL in a general way (Jafarigohar and Mortazavi, 2013; Chang et al., 2015), and others, in specific ways: notably, by boosting organizational skills (Chang and Lin, 2014), critical reflection (Sudirman et al., 2021), and critical thinking (Ahmed, 2020). Alongside these broadly positive findings about reflective-journal writing, however, a recent study by Akhmedjanova and Moeyaert (2022) reported that a sample of eight Southeast Asian students did not value the use of SRL journals in the process of learning and writing English, and that at least five of them considered the SRL journal assignments to be annoying and boring. This further highlights the importance of taking account of contextual variation when examining learners’ self-regulated writing strategy uptake (Inan-Karagul and Seker, 2021).

As briefly noted above, individual variation, notably in proficiency levels, also needs to be considered. For example, in a study by Farahian et al. (2021), an experimental group that used reflection sheets did not exhibit a significantly higher level of critical reflection than a control group. The authors speculated that learners’ L2 proficiency levels could have played a role in limiting their use of higher levels of reflection. Similarly, Yabukoshi (2020) asked four EFL learners to document their SRL processes in reflective journals and found that while the two high achievers’ writings demonstrated high self-regulation skills, good self-motivation, and adaptive decisions about future plans, those of the two low achievers were less likely to include reflection on their learning experiences or to identify their problems. Subsequently, Akhmedjanova and Moeyaert (2022) reported that their only student participant who understood key aspects of SRL-related journal writing or its value also scored highest on the baseline essay. This finding provided further evidence of a potential relationship between pre-existing proficiency and self-regulated writing strategy use while writing reflective journals. However, given the above-cited studies’ very small sample sizes, more research should be conducted to shed light on this possible relationship.

Another under-explored issue arises from the fact that most previous studies have treated reflective-journal writing simply as a means to an end, rather than investigating how students demonstrate and develop their awareness of different types of SRL strategies while engaged in it. The few exceptions notably include Yabukoshi (2020), who used reflective journals as evidence of L2 students’ individual variation in metacognitive awareness. However, that study’s analysis of SRL focused disproportionately on its metacognitive side, and on phases rather than on strategies. Another exception, Baek (2019), showed that affective learning strategies were used most frequently, and memorization strategies, least frequently, in students’ reflective journals. However, Baek’s context was EFL reading, not writing, and the coding scheme did not fully represent the SRL framework. More recently, Akhmedjanova and Moeyaert (2022) coded SRL in students’ reflective journals to show how different aspects of it (i.e., goal-setting, task management, progress monitoring, and reflection) differed before and after an SRL intervention; but again, a full picture of self-regulated writing strategy use cannot be provided by just those four areas. Lastly, Teng (2022) utilized reflective journals as a source of data for exploring EFL learners’ perceptions of a formative assessment in which an SRL intervention was embedded. The results suggested that EFL students reported increased levels of self-regulated behaviors. In particular, Teng adopted a combination of bottom-up and top-down coding approaches to explore how students engaged with the SRL intervention in terms of the relative value they placed on goal-setting, peer learning, feedback-handling, self-assessment, and reflection on their performance. But in any case, studies investigating how students demonstrate and develop their use of self-regulated writing strategies in reflective journals have been quite limited in terms of both amount and perspectives, suggesting the need for more exploration toward a comprehensive picture.

Based on the above review of the existing literature, we asked the following research questions:

RQ1. When they write reflective journals, how do students demonstrate their use of self-regulated writing strategies, and does such strategy use change over time?
RQ2. How do students with varied writing-proficiency levels differ from one another in terms of their use of self-regulated writing strategies when writing reflective journals?
RQ3. Does the use of reflective journals affect students’ perceptions of their own self-regulated writing strategies and/or L2 writing?

## Methodology

### Participants and context

This study took place in an undergraduate course at a top Chinese university during the fall semester of 2021. A total of 38 English majors participated in this research with informed consent. Among them, there were 22 females (57.9%) and 16 males (42.1%), ranging in age between 17 and 21 (*M* = 18.16, SD = 0.89). Because most of them did not have standardized writing-test scores (e.g., TOEFL or IELTS) for reference, they were asked to rate their own English-writing proficiency on a five-point scale, ranging from “1” = “very poor” to “5” = “very good.” Their average self-rated proficiency was 3.03 (SD = 0.75). Before participating in this study, none of them had received any self-regulated strategy instruction. The course, titled *English Writing and Critical Thinking*, lasted 15 weeks. The class met twice a week for 90 min each time. It was aimed at equipping its students with the basic skills of critical thinking and enabling them to use such skills in English academic writing. Over the semester, the students were required to submit five writing assignments. In the first assignment, they were expected to write a one- to two-page introduction to an essay. The second was an annotated bibliography of three to five references they planned to use in the same essay. The third assignment was a detailed outline of the essay, showcasing the structure of each of its parts as well as the claim(s), subclaim(s), and evidence that the writer intended to use. The fourth was a six- to eight-page draft of the essay developed on the basis of the outline. The final course assignment was the eight- to 10-page essay itself. In addition, there were three peer-review sessions held after the submission of the second, third, and fourth assignments, respectively. In them, students were divided into groups according to their essay topics and offered feedback to their fellow group members on conventions, content, and composition.

### Procedures

Our data-collection procedures are presented in Table 1. Data were collected from questionnaires and reflective journals. At the beginning of the semester, students filled out a background information questionnaire and a self-regulated writing strategy use questionnaire. In Week 7, they filled out the self-regulated writing strategy use questionnaire again and wrote the first reflective journal. In Week 11, they submitted the second reflective journal, after finishing the second and the third assignments and experiencing two peer review sessions. In Week 15, the students completed the self-regulated writing strategy use questionnaire for a third time. Two weeks later, they submitted their final reflective journal after completing all of their coursework.

**Table 1:** The timeline of data collection.

Time	Assignment	Peer review	Questionnaire	Reflective journal
Week 1			The background information questionnaire and the self-regulated writing strategy use questionnaire	
Week 6	Assignment 1 (introduction)			
Week 7			The self-regulated writing strategy use questionnaire	The first reflective journal
Week 8	Assignment 2 (planned references)			
Week 9		First peer review		
Week 10	Assignment 3 (outline)			
Week 11		Second peer review		The second reflective journal
Week 13	Assignment 4 (essay draft)			
Week 14		Third peer review		
Week 15			The self-regulated writing strategy use questionnaire	
Week 17	Assignment 5 (final essay)			The third reflective journal

### Instruments

#### Questionnaire

The questionnaire used in this study had two parts. The first part comprised five items covering the respondent’s name, ID number, age, gender, and self-rated English-writing proficiency. These items were aimed at offering an overview of the participants’ background information.

The second part consisted of 35 items on self-regulated writing strategy use, all responded to on the same five-point Likert scale ranging from 1 = “strongly disagree” to 5 = “strongly agree” (Appendix I). The 35 items collectively covered four dimensions: cognition, metacognition, social behavior, and motivational regulation. The 21 items that measured the first three of those dimensions were adapted from Teng and Zhang (2016a) Writing Strategies for Self-regulated Learning Questionnaire (WSSRLQ). It should be noted that *course memory*, one of the two cognitive subcategories in the original questionnaire, was not included, because it was irrelevant to the design of the current study’s focal course. Motivational regulation was measured using the 14-item L2 Writing Strategies for Motivational Regulation Questionnaire (L2WSMRQ), adapted from Teng and Zhang (2016b). In this case, *environmental structuring*, the last subcategory in the original questionnaire, was not pertinent to the focal course’s design and was therefore removed.

As the questionnaire was filled out by the participants three times to obtain process information about their self-regulated writing-strategy development, the internal reliability of each subcategory was also tested at those three time points (T1 = Week 1, T2 = Week 7, and T3 = Week 15). The results of those tests are presented in Table 2. It should be noted that although most of the scales at different time points reached a satisfactory level (i.e., larger than 0.70; Tavakol and Dennick, 2011), some scales showed low reliability. For example, the reliability of emotional control was low at three time points, and mastery self-talk was low at T1. We postulate that this may be due to the low number of items (i.e., three items) of the measured scale, which, as Pallant (2020) pointed out, could easily lead to low Cronbach values.

**Table 2:** Reliability of the questionnaire at three time points.

Category	Subcategory	Items	Cronbach α		
			T1	T2	T3
Cognitive	TP	5	0.85	0.8	0.83
Metacognitive	IP	3	0.71	0.75	0.76
	GM	6	0.75	0.82	0.86
Behavioral	FH	4	0.69	0.79	0.72
	PL	3	0.87	0.6	0.8
Motivational	MS	3	0.5	0.81	0.79
	PS	4	0.8	0.83	0.86
	IE	4	0.62	0.7	0.74
	EC	3	0.64	0.66	0.64

TP, text processing; IP, idea planning; GM, goal-oriented monitoring; FH, feedback handling; PL, peer learning; MS, mastery self-talk; PS, performance self-talk; IE, interest enhancement; and EC, emotional control.

#### Reflective journals

The students received general guiding questions (Appendix II) before each reflective journal writing session to help ensure that their journal content would be relevant but not restricted. The first two reflective journals, hereafter J1 and J2, were based on the first and the third assignments, respectively. Therefore, their guiding questions focused on changes between the first drafts and the submitted versions of these assignments; the strengths and weaknesses of their work; and their plans for future improvement. The last reflective journal, J3, was a reflection not only on the final essay but also on the learning process throughout the semester, and the guiding questions were tailored to both purposes. To better capture their experiences and feelings, the students were encouraged to write in their native language, with no limitation on word count. Those non-English entries were translated into English for report by the second, third, and fourth authors, and the first author checked all translation for accuracy. Students were assured that their journals’ content would not be rated or influence their course grades in any way, and only the researchers have access to their work. To avoid potential researcher bias and for reference in this research, the journal data were anonymized by replacing name information with an ID number ranging between 1 and 38. Because two participants failed to submit their final reflective journal entries, 112 were collected: i.e., 38 in the first round, 38 in the second, and 36 in the third.

#### Writing scores

The final scores students received on their essays were used to determine their writing proficiency levels for further analysis. The scores were given by the course instructor according to students’ performance in five dimensions: disciplinary content understanding, quality of argument, use of sources, responsiveness to the question, and clarity/focus of the writing. The theoretical maximum score for each essay was 100 and the minimum was 0.

### Data analysis

To answer our first research question, the second and third authors familiarized themselves with the reflective journal entries by reading them several times prior to coding. The entries were then coded using a combination of top–down and bottom–up approaches and continuously checked against the theoretical framework developed by Teng and Zhang (2016a) and Zhang and Zou (2022a, 2022b). Every time an act of strategy use was identified in a journal, it would be counted as one time of application for the corresponding strategy (or strategies, as sometimes one act might reflect multiple strategies). The total number of use for every strategy would be recorded for further analysis. If the same act was mentioned repeatedly, it would be recorded only once, so it was a count of “acts” rather than “accounts,” unaffected by how many words or details used for description. To ensure the credibility of coding, the same two researchers first coded the journal entries independently and then discussed their decisions with each other and with the first author, until all disagreements were resolved. The final coding scheme can be found in Appendix III. Since the data were not normally distributed, Friedman tests were conducted to explore whether the students’ use of self-regulated writing strategies changed over the semester. To further pin down when the differences actually occurred, the data collected from the three reflective journals were compared pairwise using sign tests. A Bonferroni adjustment was made to the probability criterion of statistical significance (*p* < 0.05/3 ≈ 0.017).

To answer our second research question, the students were divided into high-, medium-, and low-proficiency groups based on the final scores they received on their essays. The top 25% (10 students) and bottom 25% (10 students), respectively, made up the high-proficiency and low-proficiency groups (as per Sun et al., 2021). The remaining 50% belonged to the medium-proficiency group (18 students). The overall use of self-regulated writing strategies exhibited in reflective journals by the high- and low-proficiency groups were then compared using Mann–Whitney U tests, due to the non-normal data distribution.

To answer our final research question, quantitative and qualitative analyses were conducted. The quantitative part used one-way repeated-measures analysis of variance (ANOVA) on the questionnaire data to compare the differences in self-perceived self-regulated writing strategies at three time points. The data met the assumptions of normal distribution.

The results of Mauchly’s test suggested that all variables except for peer learning (Mauchly’s *W* = 0.78, *p* = 0.01, Greenhouse–Geisser = 0.82, Huynh–Feldt = 0.85) violated the assumption of sphericity. Thus, for the peer-learning variable, the Huynh–Feldt correction instead of F-ratio was used in the relevant one-way repeated-measures ANOVA (Field, 2009). To further determine when significant differences occurred in the measured SRL strategy variables, *post-hoc* tests with Bonferroni adjustment (*p* < 0.05/3 ≈ 0.017) were conducted.

Our qualitative analysis chiefly addressed students’ perceptions of the use of reflective journals according to their statements in the final reflective journal. The journal entries were read carefully by the third and the fourth authors. Then, initial open codes were allocated to the recurrent expressions of a given idea. After identifying the open codes, the researchers used axial coding and grouped the codes into the following 14 topical categories: summary, review, self-talk, self-recognition, reminder, record, improvement, inspiration, envisioning, feeling of accomplishment and satisfaction, willingness to keep on writing and make progress, strengthening writing techniques, instructor connection, and negative comments. In the final stage, the researchers used selective coding to merge the recognized categories into six distinct themes (see Appendix IV for the coding scheme and Appendix V for samples of students’ reflective journals).

## Results

### Use and change of self-regulated writing strategies demonstrated in reflective journals

A total of 963 descriptions of self-regulated writing strategy use were identified in the 112 reflective journal entries we collected. Among the 19 kinds of strategies, *feedback handling* (22.43%), *resource management* (12.98%), *text processing* (9.45%), *emotional control* (9.45%), and *idea planning* (9.03%) were the top five most frequently seen, accounting for almost two-thirds of all occurrences of self-regulated writing strategies. A full description of the distribution can be found in Table 3.

**Table 3:** Overall frequencies of self-regulated writing strategies demonstrated in reflective journals.

Category	Subcategory	Frequency	Number of students using the strategy	Example
Metacognitive	Self-monitoring and evaluation	60 (6.23%)	26 (68.42%)	*“I also found some problems that I did not realize before, such as inaccurate expressions and flawed argument.”* (Participant 3)
	Idea planning	87 (9.03%)	30 (78.95%)	*“I planned to study the changes of female images in Chinese TV series in chronological order and argue that such changes did not alter people’s stereotypes of female.”* (Participant 3)
	Goal-setting	18 (1.87%)	13 (34.21%)	*“Although I am still unable to have perfect logic in my article, this should be a goal for me to work on, and I will continue to improve my writing ability by applying this academic writing method.”* (Participant 30)
Cognitive	Record reviewing	12 (1.25%)	8 (21.05%)	*“I read the criteria of a good research question over and over again.”* (Participant 22)
	Contribution making	51 (5.30%)	25 (49.02%)	*“Based on what was taught in class and comments from my teacher and classmates, I found the problems and set out to solve them.”* (Participant 22)
	Elaboration	10 (1.04%)	8 (21.05%)	*“The essential difference between writing essays required in high school and writing research articles is that…”* (Participant 36)
	Text processing	91 (9.45%)	34 (89.47%)	*“I shortened sentences that were too long and too complex, revised some parts that were hard to understand in the gap section, and improved some collocations.”* (Participant 12)
	Visualization	4 (0.42%)	4 (10.53%)	*“Before I started writing in earnest, I drew a mind map to help me sort the literature and information that I collected.”* (Participant 8)
	Perspective change	34 (3.53%)	24 (70.59%)	*“I realized that there was a huge gap between my understanding and readers’ understanding, and kept trying to think from readers’ perspective when writing.”* (Participant 32)
Motivational	Emotional control	91 (9.45%)	32 (84.21%)	*“At first, I felt painful and lost when I had to revise my work…but generally my revised version became better, which gave me a strong sense of achievement.”* (Participant 20)
	Effort regulation	40 (4.15%)	24 (63.16%)	*“This was a time-consuming and arduous task, but I made progress bit by bit.”* (Participant 17)
	Self-consequence	1 (0.10%)	1 (2.63%)	*“I had higher expectation of good comments because I thought I spent more time and energy, which should lead to better results.”* (Participant 33)
	Interest enhancement	13 (1.35%)	10 (26.32%)	*“However, I knew that I was very interested in this topic, so I was willing to spend a lot of time writing and searching for relevant literature.”* (Participant 2)
	Performance self-talk	3 (0.31%)	3 (7.89%)	*“When I realized that they were so smart, I worked even harder because I thought as a senior I should not underperformed them.”* (Participant 2)
	Mastery self-talk	13 (1.35%)	13 (34.21%)	*“How could I consider this essay as perfect at that moment? Obviously there are problems and room for improvement everywhere.”* (Participant 16)
Behavioral	Peer learning	65 (6.75%)	32 (84.21%)	*“But I found a fatal error after I discussed this idea with my classmates.”* (Participant 25)
	Seeking help	29 (3.01%)	18 (47.37%)	*“I had the audacity to ask my roommates and friends also majoring in English to help review my work (probably the introduction assignment).”* (Participant 20)
	Feedback handling	216 (22.43%)	38 (100.00%)	*“Based on the review by my classmates and teacher, I revised the wording in some parts of draft 1 to better express myself.”* (Participant 12)
	Resource management	125 (12.98%)	33 (86.84%)	*“I also added supporting references when needed.”* (Participant 4)

Table 4 presents the results of Friedman tests, which show statistically significant changes in the use of eight strategies: *self-monitoring and evaluation* (*X*^2^ = 9.08, *p* < 0.05), *idea planning* (*X*^2^ = 9.12, *p* < 0.05), *text processing* (*X*^2^ = 18.12, *p* < 0.001), *perspective change* (*X*^2^ = 7.58, *p* < 0.05), *emotional control* (*X*^2^ = 28.83, *p* < 0.001), *effort regulation* (*X*^2^ = 13.02, *p* < 0.01), *peer learning* (*X*^2^ = 29.96, *p* < 0.001), and *resource management* (*X*^2^ = 11.52, *p* < 0.01). For these eight strategies, the results of *post-hoc* tests (Table 5) further indicated that: (1) between J1 and J2, text processing decreased at the *p* < 0.001 level; (2) between J2 and J3, self-monitoring and evaluation increased at the *p* < 0.05 level, resource management increased at the *p* < 0.01 level, and both emotional control and peer learning increased at the *p* < 0.001 level; and (3) between J1 and J3, both idea planning and effort regulation increased at the *p* < 0.01 level, and both emotional control and peer learning increased at the *p* < 0.001 level.

**Table 4:** Results of Friedman tests regarding the use of self-regulated writing strategies identified in reflective journals.

Category	Subcategory	Median			*df*	Chi-square (*X^2^*)	*p*
		J1	J2	J3			
Metacognitive	Self-monitoring and evaluation	0	0	1	2	9.08	**0.011**
	Idea planning	1	1	0	2	9.12	**0.01**
	Goal-setting	0	0	0	2	0	1
Cognitive	Record reviewing	0	0	0	2	4.75	0.093
	Contribution making	0	0	0	2	0.18	0.913
	Elaboration	0	0	0	2	2	0.368
	Text processing	1	0	1	2	18.12	**0**
	Visualization	0	0	0	2	0	1
	Perspective change	0	0	0	2	7.58	**0.023**
Motivational	Emotional control	0	0	2	2	28.83	**0**
	Effort regulation	0	0	1	2	13.02	**0.001**
	Self-consequence	0	0	0	2	2	0.368
	Interest enhancement	0	0	0	2	1.75	0.417
	Performance self-talk	0	0	0	2	2	0.368
	Mastery self-talk	0	0	0	2	4.77	0.092
Behavioral	Peer learning	0	0	1	2	29.96	**0**
	Seeking help	0	0	0	2	1.36	0.507
	Feedback handling	2	1	2	2	4.37	0.112
	Resource management	1	1	0	2	11.52	**0.003**

The bold values are statistically significant results.

**Table 5:** Results of *post-hoc* tests regarding the use of self-regulated writing strategies identified in different reflective journals of all students.

Category	Subcategory	J1 vs. J2		J2 vs. J3		J1 vs. J3	
		*Median difference*	*p*	*Median difference*	*p*	*Median difference*	*p*
Metacognitive	Self-monitoring and evaluation	0	0.077	−1	**0.013**	–1	0.21
	Idea planning	0	1	1	0.031	1	**0.007**
Cognitive	Text processing	1	**0**	–1	0.043	0	0.054
	Perspective change	0	1	0	0.064	0	0.031
Motivational	Emotional control	0	0.824	−2	**0**	−2	**0**
	Effort regulation	0	1	−1	0.019	−1	**0.004**
Behavioral	Peer learning	0	0.549	−1	**0**	−1	**0**
	Resource management	0	1	1	**0.001**	1	0.023

Adjustment for multiple comparisons: Bonferroni. The bold values are statistically significant results.

### Differences in the use of self-regulated writing strategies between the high- and low-proficiency groups

As shown in Table 6, Mann–Whitney U tests revealed significant differences between the high- and low-proficiency groups in their use of four self-regulated writing strategies as demonstrated in their reflective journals. These were: *idea planning* (*Z* = −2.46, *p* < 0.05), *goal-setting* (*Z* = −2.01, *p* < 0.05), *feedback handling* (*Z* = −2.32, *p* < 0.05), and *resource management* (*Z* = −4.16, *p* < 0.001). Specifically, the high-proficiency group applied *idea planning*, *feedback handling*, and *resource management* significantly more often than the low-proficiency group did, with mean rank differences of 10.14, 10.14, and 17.74, respectively. The low-proficiency group, in contrast, tended to use *goal-setting* significantly more frequently, with a mean rank difference of 5.04.

**Table 6:** Results of Mann–Whitney *U* tests regarding the use of self-regulated writing strategies identified in reflective journals by high- and low-proficiency groups.

Category	Subcategory	Mean rank		*Z*	*p*
		High proficiency	Low proficiency		
Metacognitive	Self-monitoring and evaluation	30.82	30.18	−0.17	0.866
	Idea planning	35.57	25.43	−2.46	**0.014**
	Goal-setting	27.98	33.02	−2.01	**0.045**
Cognitive	Record reviewing	31	30	−0.4	0.69
	Contribution making	32.83	28.17	−1.2	0.231
	Elaboration	30.5	30.5	0	1
	Text processing	30.53	30.47	−0.02	0.987
	Visualization	30	31	−1	0.317
	Perspective change	30.5	30.5	0	1
Motivational	Emotional control	31.95	29.05	−0.69	0.488
	Effort regulation	30.77	30.23	−0.15	0.878
	Self-consequence	30.5	30.5	0	1
	Interest enhancement	31.5	29.5	−1.43	0.154
	Performance self-talk	29.5	31.5	−1.43	0.154
	Mastery self-talk	29.5	31.5	0.75	0.451
Behavioral	Peer learning	31.1	29.9	−0.3	0.768
	Seeking help	32.17	28.83	−1.03	0.302
	Feedback handling	35.57	25.43	−2.32	**0.021**
	Resource management	39.37	21.63	−4.16	**0**

The bold values are statistically significant results.

### The relationship between writing reflective journals on self-regulated writing strategies and L2 writing

Table 7 presents the descriptive statistics and results of one-way repeated-measures ANOVA of change over time in the participants’ self-perceived self-regulated writing strategy use reflected in the questionnaire data. The use of reflective journals was associated with statistically significant increases in the mean scores assigned to the following four strategies: *idea planning* (*p* < 0.001), *goal-oriented monitoring* (*p* < 0.05), *peer learning* (*p* < 0.01), and *interest enhancement* (*p* < 0.001). Results of *post-hoc* tests (Table 8) indicated that, for *idea planning*, the statistically significant changes occurred between T1 and T3, with a mean difference of 0.36 (*p* < 0.001), and between T2 and T3, with a mean difference of 0.22 (*p* < 0.05). For *peer learning*, the statistically significant change occurred between T2 and T3, with a mean difference of 0.34 (*p* < 0.05); and for *interest enhancement*, such changes occurred between T1 and T3, with a mean difference of 0.30 (*p* < 0.01), and between T2 and T3, with a mean difference of 0.36 (*p* < 0.001). However, for *goal-oriented monitoring*, no statistically significant change can be said to have occurred. In other words, as compared with the first half of the semester, there was an accelerated increase in the students’ use of *idea planning*, *peer learning* and *interest enhancement* in the second half.

**Table 7:** Descriptive statistics and results of one-way repeated ANOVA tests on self-perceived self-regulated writing strategies reflected in questionnaires.

Category	Subcategory	T1			T2			T3			*F*	*p*
		*M*	95% CI	SD	*M*	95% CI	SD	*M*	95% CI	SD		
Cognitive	TP	4.23	[4.03, 4.43]	0.6	4.35	[4.21, 4.50]	0.44	4.45	[4.29, 4.61]	0.49	2.99	0.056
Metacognitive	IP	4.24	[4.07, 4.41]	0.52	4.38	[4.21, 4.55]	0.52	4.6	[4.45, 4.75]	0.46	8.96	**0**
	GM	4.02	[3.88, 4.16]	0.43	3.96	[3.76, 4.15]	0.59	4.18	[3.97, 4.39]	0.63	3.98	**0.023**
Behavioral	PL^*^	3.95	[3.65, 4.24]	0.9	3.99	[3.78, 4.20]	0.65	4.33	[4.12, 4.55]	0.64	6.09	**0.006**
	FH	4.47	[4.30, 4.64]	0.51	4.45	[4.29, 4.60]	0.47	4.61	[4.46, 4.77]	0.47	3.11	0.051
Motivational	IE	4.18	[4.00, 4.37]	0.55	4.13	[3.95, 4.32]	0.56	4.49	[4.34, 4.64]	0.45	13.27	**0**
	MS	4.45	[4.30, 4.59]	0.45	4.35	[4.19, 4.52]	0.5	4.46	[4.29, 4.63]	0.52	1.08	0.344
	EC	4.18	[4.01, 4.36]	0.53	4.11	[3.91, 4.32]	0.63	4.23	[4.03, 4.42]	0.6	0.63	0.534
	PS	3.91	[3.69, 4.14]	0.7	3.73	[3.50, 3.96]	0.71	3.92	[3.67, 4.18]	0.78	2.09	0.131

TP, text processing; IP, idea planning; GM, goal-oriented monitoring; PL, peer learning; FH, feedback handling; IE, interest enhancement; MS, mastery self-talk; EC, emotional control; and PS, performance self-talk.

^*^For PL, Mauchly’s test indicated that the assumption of sphericity has been violated, *χ*^2^(2) = 9.03, *p* < 0.05. Therefore degree of freedoms were corrected using Huynh–Feldt estimates of sphericity (*ε* = 0.85). The bold values are statistically significant results.

**Table 8:** *Post-hoc* tests of the mean difference on self-perceived self-regulated writing strategies reflected in questionnaires.

	T1 vs. T2			T1 vs. T3			T2 vs. T3		
	Mean difference	95% CI	*p*	Mean difference	95% CI	*p*	Mean difference	95% CI	*p*
IP	−0.14	[−0.39, 0.11]	0.49	−0.36	[−0.57, −0.15]	**0**	−0.22	[−0.40, −0.04]	**0.014**
GM	0.06	[−0.14, 0.26]	1	−0.16	[−0.38, 0.05]	0.204	−0.22	[−0.42, −0.03]	0.022
PL	−0.04	[−0.36, 0.27]	1	−0.39	[−0.74, −0.03]	0.03	−0.34	[−0.57, −0.11]	**0.002**
IE	0.05	[−0.12, 0.23]	1	−0.3	[−0.49, −0.12]	**0.001**	−0.36	[−0.56, −0.15]	**0**

IP, idea planning; GM, goal-oriented monitoring; PL, peer learning; and IE, interest enhancement. Adjustment for multiple comparisons: Bonferroni. The bold values are statistically significant results.

Our qualitative analysis of the participants’ perceptions of reflective-journal writing revealed that in general, they held a positive attitude toward it. First, they perceived such writing as a way of promoting self-monitoring and evaluation. To be more specific, they perceived it as a means of: (1) summarizing, for example, “*Reflective writing helps me summarize what I have learnt in the process of writing and revising, which would benefit my future writing”* (Participant 24); (2) reviewing, for example, “*Reflective writing enables me to look back on what I have learnt and record my personal growth in a timely manner*” (Participant 8); (3) engaging in self-talk, for example, “*Reflective writing is like a dialog with myself*” (Participant 30); (4) engaging in self-recognition, for example, “*Reflective writing gives me a chance to evaluate strengths and weaknesses in my writing*” (Participant 31); (5) reminding, for example, “*Reflective writing reminds me to focus on my problems in writing and push me to think how I can do better next time*” (Participant 16); and (6) recording, for example, “*In reflective writing, I would write down my thoughts before, during, and after writing my essays*” (Participant 20).

Second, these students perceived reflective writing as a process of goal-setting and future idea planning. This included: (1) writing improvement, for example, “*I always review my reflective writings before starting to write in order to make improvements and avoid past problems*” (Participant 16); (2) inspiration, for example, “*I am greatly inspired by reflective writing, from which I learn techniques and gain motivation for my future writings*” (Participant 25); (3) envisioning, for example, “*Through reflective writing, I envision future studies in writing*” (Participant 4); and (4) extension, for example, “*Reflective writing not only helps me in writing, but in every other course I am taking*” (Participant 10).

Third, the participants perceived reflective writing as a way of stimulating their motivation. Through writing reflective journals, they said, they gained (1) a sense of accomplishment and satisfaction, for example, “*Reflective writing helps me polish my essays, which gives me a sense of accomplishment*” (Participant 20) and (2) willingness to keep on writing and make progress, for example, “*Through reflective writing, I realize that many problems are not as difficult to solve as I expected. I am willing to find out the root cause and solve it*” (Participant 36).

Fourth, they perceived reflective writing as a way of strengthening their writing techniques. One examples of this perception was “*Through reflective writing, I pay close attention to my language, logic, and supporting facts. My writing greatly improves after my reflection*” (Participant 27). The improvement in writing techniques was “*systematic*” (Participant 10), as Participant 27 put it: “*The awareness of constantly paying attention to logical coherence and using facts to support points of view has been deeply engrained in every piece of writing of mine.*”

Finally, the participants perceived reflective writing as a way of establishing connections with and gaining support from their teachers, for example, “*Reflective writing enables me to create a close connection with my teacher. I have learnt a lot from her feedback on my reflective writing. She also encourages me a lot*” (Participant 7). Reflective writing was especially useful when they were challenged with negative feelings. “*I told my teacher about my anxiety and confusion through reflective writing and felt very lucky to get encouragement and suggestions from her,*” wrote Participant 25.

Although the majority of the sampled students expressed positive thoughts and feelings about reflective-journal writing, some responses characterized it as “*time-consuming*” (Participant 17), “*causing anxieties*” (Participant 11), and even “*miserable*” (Participant 18). Some felt that this activity had barely any effect on the quality of their writing, for example, “*I still cannot guarantee I’ll get a higher score next time*” (Participant 35), and that “*Even if I am aware of my writing problems, I may make mistakes again in my next writing assignment. Reflective writing is not an easy way to completely correct my mistakes*” (Participant 33).

## Discussion

### Use and change of self-regulated writing strategies as demonstrated in reflective journals

Some previous questionnaire-based studies have suggested a moderate-to-high level of self-regulated writing strategy use (e.g., Abadikhah et al., 2018; Mbato and Cendra, 2019; Bai and Guo, 2021). The present study found more variations: among the 19 kinds of strategies identified in reflective-journal writing, only five (i.e., *handling feedback*, *resource management*, *text processing*, *emotion regulation*, and *idea planning*) were demonstrated relatively frequently, accounting for almost two thirds of all appearances of self-regulated writing strategies. On one hand, this indicates the special link between reflective-journal writing and SRL, as evidenced by the fact that all four types of self-regulated writing strategies (i.e., cognitive, metacognitive, behavioral, and motivational) are reflected (e.g., Teng and Zhang, 2016a, 2018; Csizér and Tankó, 2017; Hu and Gao, 2018). On the other, it suggests a disproportionate prioritization of some types of strategies (e.g., *handling feedback*) over others.

Interestingly, the above results contradict Xu’s (2021) finding that Chinese EFL writers adopted fewer social-behavior strategies than other self-regulated writing strategies. A possible explanation for this discrepancy is that the choice of social-behavior strategies is closely linked to specific guidance provided by English teachers (Bai et al., 2020). In our study, the teacher placed a high value on article revision, and the course’s second and subsequent writing assignments depended heavily on previous ones. These distinctive course features both led to the students making strong efforts to address how they handled feedback from the teacher and their peers. In addition, they used reflective-journal writing as a major channel for conversing with the teacher as well as with themselves, implying the dialogic nature of such writing from a sociocultural perspective (Rassaei, 2015). On the whole, we believe this is a positive sign about reflective writing, i.e., that it pushes students both to think critically, and to deal carefully with the feedback they receive (Thorpe, 2004; Ahmed, 2020; Sudirman et al., 2021) even if they do not fully understand it (Zhang and Zou, 2022a, 2022b). At the same time, however, we argue that—given the overall key role of SRL in determining how students initiate, sustain, regulate and monitor their L2 learning (Dörnyei, 2005; Tseng et al., 2006), teachers designing guidance for reflective-journal writing should pay special attention to those strategies that were infrequently used by our participants, that is, strategies which learners may be unfamiliar with and unable to use. Such explicit strategy instruction would be beneficial as regulation may benefit from shifting from being self-regulation to co/other-regulation, so that students are prepared to be successful self-regulated learners (Thomas and Rose, 2019; Thomas et al., 2021).

Regarding change over time in the use of self-regulated writing strategies, our journal data firstly suggest that not all such strategies grew significantly. This echoes previous findings (Jackson and Park, 2020; Wilby, 2020) and further confirms the complex nature of the use of self-regulated writing strategies in the real world (Oxford, 2017). Secondly, these data show that seven strategies (*self-monitoring and evaluation*, *idea planning*, *perspective change*, *emotional control*, *effort regulation*, *peer learning*, and *resource management*) did significantly increase over time, and most such changes occurred during the second half of the semester, adding empirical evidence to the existing literature regarding such improvement (Han and Hiver, 2018; Sasaki et al., 2018). Importantly, these seven strategies reflected all four aspects of self-regulated writing (i.e., cognitive, metacognitive, behavioral, and motivational), lending support to the idea that reflective writing can promote SRL-related strategies (e.g., Jafarigohar and Mortazavi, 2013; Chang et al., 2015). And thirdly, we found that *text processing* decreased significantly from J1 to J2. In this study, *text processing* was defined as *using linguistic knowledge* (e.g.*, grammar, spelling, punctuation, clear expression*) *to revise or improve writing texts*, so our finding could be explained by the fact that novice L2 writers usually pay more attention to “mechanics rather than content” (Mu and Carrington, 2007, p. 10) and are relatively lacking in knowledge of the generic, discourse, and rhetorical aspects of academic writing (e.g., Swales, 2004; Lillis and Curry, 2010). As the course progressed, the students received an increasing amount of overt instruction in generic, discourse, and rhetorical moves (e.g., Cargill et al., 2018; Li and Flowerdew, 2020), and this could explain why less *text processing* was found in the latter half of the course.

### Differences in the use of self-regulated writing strategies between the high- and low-proficiency groups

Some previous studies on the use of reflective writing have tended to hypothesize a potential influence of individual proficiency levels on observable SRL-related behaviors (Farahian et al., 2021), while others argued that such a link exists while treating SRL strategy use as a holistic construct (Yabukoshi, 2020; Akhmedjanova and Moeyaert, 2022). Our study has not only revealed the influence of individual-proficiency variation on the use of self-regulated writing strategies when writing reflective journals, but also paints a clear picture of where such variation lies. First, we found that our high-proficiency group applied *idea planning*, *feedback handling*, and *resource management* significantly more often than the low-proficiency group did. This clearly reflects the critical role of the three major processes of L2 writing, i.e., planning, composing, and reviewing (Flower and Hayes, 1981; Hayes, 1996), and especially how “before writing,” “during writing,” and “after writing” strategies may all contribute to writing. This finding extends, to a new context, previous ones regarding how expert writers outperform novice ones in planning (e.g., Chien, 2012; Bai et al., 2020, 2022; Guo and Bai, 2022). However, our finding regarding the roles of *feedback handling* and *resource management* in differentiating students do not appear to have been reported in any previous study. We believe this finding could add weight to arguments in favor of the use of reflective journals to promote SRL strategy-use behavior, over and above this practice’s known cognitive and metacognitive advantages (Thorpe, 2004; Ahmed, 2020; Sudirman et al., 2021). The same finding may also have important pedagogical implications for L2 teachers who design, assign, and assess reflective journals, as their support could be important for students on their way toward successful self-regulated learners (Thomas and Rose, 2019; Thomas et al., 2021). In particular, for those learners who have difficulties in writing these journals in particular, additional scaffolding for *idea planning*, *feedback handling*, and *resource management* should be put in place.

Additionally, we found that while high-proficiency students reported significantly more *idea planning* strategies in their reflective-journal entries than their low-proficiency counterparts did, the former demonstrated significantly less *goal-setting* than the latter. A clarification of these two constructs could help explain this apparent contradiction. Unlike Sun and Wang (2020), who combined them under the label *goal-setting and idea planning*, we found them to be clearly distinguishable from each other in our data. That is, *goal-setting* involved setting more abstract goals and sub-goals of learning, for example, “*Although I am still unable to have perfect logic in my article, this should be a goal for me to work on, and I will continue to improve my writing ability by applying this academic writing method*” (Participant 30). *Idea planning*, on the other hand, was more specific, executable and task-oriented, for example, “*I planned to study the changes in female images in Chinese TV series in chronological order and argue that such changes did not alter people’s stereotypes of females*” (Participant 3). In this context, it should also be noted that *idea planning* and *goal-setting* as captured in our study were consistent with how those two constructs were conceptualized in some previous research (e.g., Zhang, 2010). For example, in Teng and Zhang’s (2016a) WSSRLQ, one example given for *idea planning* is “*Before writing, I read related articles to help me plan*,” whereas an example of *goal-oriented monitoring and evaluating* is “*When learning to write, I set up goals for myself in order to direct my learning activities*” (p. 700). It may also be important that, although the arrangement of personal goals is vital in SRL (Zimmerman and Schunk, 2008, 2011), only goals that incorporate specific performance standards, are proximal, and are of a reasonable difficulty level are widely considered ‘good’ goals (Locke et al., 1981; Bandura, 1988). Such goals promote performance by focusing on the amount of effort required for success and self-anticipation (Schunk, 1990). Given that most of the goals we identified in our journal data were rather broad and vague, it is perhaps unsurprising that low-proficiency students demonstrated significantly more use of *goal-setting* than high-proficiency students did.

### The use of reflective-journal writing

The qualitative results suggest that the practice of journaling raised students’ awareness and may have contributed to an increase in their self-regulated writing strategy use. This finding is consistent with a number of previous studies regarding the positive role of reflective writing in L2 writing instruction (e.g., Chang and Lin, 2014; Chang et al., 2015; Rassaei, 2015; Wu and Lin, 2015; Hussein, 2018; Baek, 2019; Hussein et al., 2020; Farahian et al., 2021; Lv et al., 2021; Sudirman et al., 2021). In addition, our participants reported that reflective journaling not only helped to strengthen their writing techniques, but also made them feel more connected to their teacher. From a sociocultural point of view, this finding illustrates a co-regulated language learning mode to be explored (Gao and Hu, 2020; Thomas et al., 2021) and highlights the importance of encouraging students to have conversations with themselves and with the teacher (Rassaei, 2015) as a means of gaining new knowledge (Moon, 2006; Chang and Lin, 2014; Hussein, 2018; Baek, 2019; Hussein et al., 2020; Sudirman et al., 2021). Our findings augment the literature by showing how reflective journals may create a space for (1) developing one’s goal-setting and idea-planning ability; (2) enhancing one’s interest in writing subsequent assignments; and (3) fostering one’s ability to learn from peers. These findings highlight how crucial reflective journaling could be to SRL’s self-reflection phase (Zimmerman, 2000) and all four aspects of L2 writing (e.g., Teng and Zhang, 2016a, 2018; Csizér and Tankó, 2017; Hu and Gao, 2018). In addition, our findings suggest that reflective writing is a socio-cultural activity (Rassaei, 2015), which (1) is capable of facilitating the formation of writing communities (Graham, 2018), (2) treats writing as a process rather than a product (Murray, 1972), and (3) deepens teacher-student relationships (Lee and Schallert, 2008). All of these factors seem likely to contribute positively to various aspects of students’ self-regulated writing strategy growth.

On the other hand, the sporadic negative comments we received on reflective writing echoed Akhmedjanova and Moeyaert’s (2022) findings of students failing to recognize the value of writing SRL journal, despite the relatively lower burden of our reflective-writing assignments. A possible explanation could be that the students who complained were relatively lacking in declarative knowledge about SRL strategies (Mallahi, 2020; Sun and Wang, 2020; Akhmedjanova and Moeyaert, 2022), and perhaps especially about the power of reflection in writing (e.g., Hussein, 2018; Baek, 2019; Farahian et al., 2021; Sudirman et al., 2021). If so, they would have been less likely to access these mental resources while composing both reflective writing and writing assignments. In addition, students’ individual-difference factors such as their attitudes toward and experience of reflective writing (Atkinson, 2016) could also have led to negative perceptions of our journal-writing assignments. However, the existence of these few negative comments should not be taken as detracting from the participants’ overall positive perceptions of reflective-journal writing.

## Conclusion

To maximize the benefits of self-reflection, many L2 educators require their students to write reflective journals (e.g., Takeuchi, 2019; Sudirman et al., 2021), but the relationship between this practice and self-regulated writing strategy use has long remained unclear. To help clarify this issue, the present study began by tracing how students demonstrated and developed their awareness of different types of SRL strategies when writing reflective journals. It found such demonstration and its development to be uneven. Specifically, *handling feedback* was exhibited the most in students’ reflective journals, and seven strategies (*self-monitoring and evaluation, idea planning, perspective change, emotional control, effort regulation, peer learning, and resource management*) showed significant increase over time, especially during the second half of the semester. In addition, we identified considerable variation in SRL use by writing-proficiency levels, with highly proficient students applying *idea planning*, *feedback handling*, and *resource management* and low-proficiency students applying *goal-setting*, significantly more often than the other group did. Furthermore, the qualitative results of the current study provide evidence about the potential effectiveness of using reflective-journal writing to promote self-regulated writing strategies.

Pedagogically, this work’s most important implication is that teachers should consider using reflective journaling as a powerful tool for SRL development. They should purposefully design structures for reflective writing that cover the cognitive, metacognitive, motivational and behavioral aspects of their students’ learning. Specifically, given the uneven distribution of SRL-strategy adoption, both across students and across contexts, it is necessary for teachers to insert appropriate guiding questions to scaffold students’ reflection, rather than merely asking them to reflect. Second, reflective journaling should be clearly defined as a process rather than a product. That is, assigning journal writing at multiple time-points could help develop students’ awareness of self-regulated writing strategies gradually and steadily. Also, these tasks should be assigned in reasonable quantities and at reasonable intervals, to avoid students becoming anxious about them, annoyed, or bored. And fourth, teachers should use reflective-journal writing as an opportunity to facilitate more individualized SRL guidance. For instance, when guiding low-proficiency students in this activity, they could offer explicit instruction in how any goals expressed should be specific, executable, and task-oriented instead of broad and generic.

This study has the following limitations. First, it only recruited English-major undergraduates from one top Chinese university, and the number of participants in the high- and low-proficiency groups was rather small; so, studying a larger number of participants from more diverse academic and ethnolinguistic backgrounds could yield different insights. Second, in the case of certain strategies such as *peer learning* and *feedback handling*, reflective journaling may not have been the only influential factor—especially due to the existence of peer-review sessions. Future studies could refine their research designs to allow clearer discernment of the reflective journals’ effects. Third, in this study we compared the differences in the use of self-regulated writing strategies between high- and low-proficiency groups as between-subjects variations. Future studies could consider testing between-subjects variation at an individual level in order to better generalize strategy use across participants. Fourth, although the qualitative findings seem to suggest that journal writing may have contributed to an increase in self-regulated writing strategy use, a causal relationship cannot be established without the inclusion of a control group (students who did not participate in reflective journaling). Fifth, our three time-points for data collection may have been few and close together to reflect the full fluctuating nature of SRL strategy use over time that previous researchers have reported. Thus, data collection at a larger number of time points across a longer overall period could yield more accurate results regarding such change.

## Data availability statement

The raw data supporting the conclusions of this article will be made available by the authors, without undue reservation.

## Ethics statement

The studies involving human participants were reviewed and approved by Tsinghua University. The patients/participants provided their written informed consent to participate in this study.

## Author contributions

YZ: conception, design, literature review, data analysis, and writing of the manuscript. ML: literature search, data collection and analysis. YC: literature search, data collection and analysis. SH: literature search and data analysis. All authors contributed to the article and approved the submitted version.

## Funding

This project was funded by the Undergraduate Education Teaching Reform Project of Tsinghua University, no. 224, Evaluating the effectiveness of integrating writing portfolios in an English academic writing course.

## Conflict of interest

The authors declare that the research was conducted in the absence of any commercial or financial relationships that could be construed as a potential conflict of interest.

## Publisher’s note

All claims expressed in this article are solely those of the authors and do not necessarily represent those of their affiliated organizations, or those of the publisher, the editors and the reviewers. Any product that may be evaluated in this article, or claim that may be made by its manufacturer, is not guaranteed or endorsed by the publisher.

## References

[ref1] Abadikhah S. Aliyan Z. Talebi S. H. (2018). EFL students’ attitudes towards self-regulated learning strategies in academic writing. Issues in Educ. Res. 28, 1–17. doi: 10.3316/ielapa.437737151804355

[ref2] Ahmed A. M. (2020). From reluctance to addiction: the impact of reflective journals on Qatari undergraduate students’ learning. Reflective Pract. 21, 251–270. doi: 10.1080/14623943.2020.1735328

[ref3] Akhmedjanova D. Moeyaert M. (2022). Self-regulated writing of English learners: intervention development. Frontiers. Education 7:841395. doi: 10.3389/feduc.2022.841395

[ref4] Alanazi M. H. (2020). The predictive effects of self-regulated writing strategies on writing performance of Saudi EFL university students. J. Educ. Psychol. Stud. 14, 668–678. doi: 10.53543/jeps.vol14iss4pp668-678

[ref5] Atkinson D. (2016). “Second language writing and culture,” in Handbook of Second and Foreign Language Writing. eds. Manchón R. M. Matsuda P. K. (Berlin: Walter de Gruyte), 545–565.

[ref6] Baek J. (2019). EFL college students’ learning experiences during film-based reading class: focused on the analysis of students’ reflective journals. Int. J. Adv. Cult. Technol. 7, 49–55. doi: 10.17703/IJACT.2019.7.4.49

[ref7] Bai B. Guo W. (2018). Influences of self-regulated learning strategy use on self-efficacy in primary school students’ English writing in Hong Kong. Read. Writ. Q. 34, 523–536. doi: 10.1080/10573569.2018.1499058

[ref8] Bai B. Guo W. (2021). Motivation and self-regulated strategy use: relationships to primary school students’ English writing in Hong Kong. Lang. Teach. Res. 25, 378–399. doi: 10.1177/1362168819859921

[ref9] Bai B. Guo W. Wang C. (2022). Relationships between struggling EFL writers’ motivation, self-regulated learning (SRL), and writing competence in Hong Kong primary schools. Appl. Linguist. Rev. doi: 10.1515/applirev-2020-0131

[ref10] Bai B. Shen B. Mei H. (2020). Hong Kong primary students’ self-regulated writing strategy use: influences of gender, writing proficiency, and grade level. Stud. Educ. Eval. 65:100839. doi: 10.1016/j.stueduc.2020.100839

[ref11] Bandura A. (1988). “Self-regulation of motivation and action through goal systems,” in Cognitive perspectives on emotion and motivation. eds. Hamilton V. Bower G. H. Frijda N. H. (Dordrecht: Kluwer Academic Publishers), 37–61.

[ref12] Cargill M. Gao X. Wang X. O’Connor P. (2018). Preparing Chinese graduate students of science facing an international publication requirement for graduation: adapting an intensive workshop approach for early-candidature use. Engl. Specif. Purp. 52, 13–26. doi: 10.1016/j.esp.2018.05.002

[ref13] Chang C.-C. Liang C. Shu K.-M. Tseng K.-H. Lin C.-Y. (2015). Does using e-portfolios for reflective writing enhance high school students’ self-regulated learning? Technol. Pedagog. Educ. 25, 317–336. doi: 10.1080/1475939X.2015.1042907

[ref14] Chang M.-M. Lin M.-C. (2014). The effect of reflective learning e-journals on reading comprehension and communication in language learning. Comput. Educ. 71, 124–132. doi: 10.1016/j.compedu.2013.09.023

[ref15] Chien S.-C. (2012). Students’ use of writing strategies and their English writing achievements in Taiwan. Asia Pacific J. Educ. 32, 93–112. doi: 10.1080/02188791.2012.655240

[ref16] Csizér K. Tankó G. (2017). English majors’ self-regulatory control strategy use in academic writing and its relation to L2 motivation. Appl. Linguis. 38, amv033–amv404. doi: 10.1093/applin/amv033

[ref17] Cumming A. (1989). Writing expertise and second language proficiency. Lang. Learn. 39, 81–135. doi: 10.1111/j.1467-1770.1989.tb00592.x

[ref18] Cumming A. Busch M. Zhou A. (2002). “Investigating learners’ goals in the context of adult second-language writing” in New Directions for Research in L2 Writing. eds. Ransdell S. Barbier M. L. (Netherlands: Kluwer), 189–208.

[ref19] Dörnyei Z. (2005). The Psychology of the Language Learner: Individual Differences in Second Language Acquisition. Hillsdale, NJ: Lawrence Erlbaum Associates.

[ref20] Farahian M. Avarzamani F. Rajabi Y. (2021). Reflective thinking in an EFL writing course: to what level do portfolios improve reflection in writing? Think. Ski. Creat. 39:100759. doi: 10.1016/j.tsc.2020.100759

[ref21] Field A. P. (2009). Discovering Statistics Using SPSS (3rd *Edn*). Thousand Oaks, CA: SAGE.

[ref22] Flower L. Hayes J. R. (1981). A cognitive process theory of writing source: college composition and communication. Coll. Compos. Commun. 32, 365–387. doi: 10.2307/356600

[ref23] Gao X. Hu J. (2020). “From language learning strategy research to a sociocultural understanding of self-regulated learning” in Autonomy in Language Education: Theory, Research and Practice. eds. Raya M. J. Vieira F. (London: Routledge), 31–45.

[ref24] Graham S. (2018). A revised writer(s)-within-community model of writing. Educ. Psychol. 53, 258–279. doi: 10.1080/00461520.2018.1481406

[ref25] Guo W. Bai B. (2022). Effects of self-regulated learning strategy use on motivation in EFL writing: a comparison between high and low achievers in Hong Kong primary schools. Appl. Linguist. Rev. 13, 117–139. doi: 10.1515/applirev-2018-0085

[ref26] Guo W. Bai B. Song H. (2021). Influences of process-based instruction on students’ use of self-regulated learning strategies in EFL writing. System 101:102578. doi: 10.1016/j.system.2021.102578

[ref27] Han J. Hiver P. (2018). Genre-based L2 writing instruction and writing-specific psychological factors: the dynamics of change. J. Second. Lang. Writ. 40, 44–59. doi: 10.1016/j.jslw.2018.03.001

[ref28] Hayes J. R. (1996). “A new framework for understanding cognition and affect in writing” in The Science of Writing: Theories, Methods, Individual Differences, and Application. eds. Levy C. M. Ransdell S. (Hillsdale, NJ: Lawrence Erlbaum Associates), 1–55.

[ref29] Hu J. Gao X. (2018). Self-regulated strategic writing for academic studies in an English-medium-instruction context. Lang. Educ. 32, 1–20. doi: 10.1080/09500782.2017.1373804

[ref30] Hussein H. (2018). Examining the effects of reflective journals on students’ growth mindset: a case study of tertiary level EFL students in the United Arab Emirates. IAFOR J. Educ. 6, 33–50. doi: 10.22492/ije.6.2.03

[ref31] Hussein H. A. R. A. Jamal D. A. H. A. Sadi I. (2020). Students’ reflective journals and creative writing in EFL. Univ. J. Educ. Res. 8, 3484–3495. doi: 10.13189/ujer.2020.080823

[ref32] Inan-Karagul B. Seker M. (2021). Improving language learners’ use of self-regulated writing strategies through screencast feedback. SAGE Open 11:215824402110648. doi: 10.1177/21582440211064895

[ref33] Jackson D. O. Park S. (2020). Self-regulation and personality among L2 writers: integrating trait, state, and learner perspectives. J. Second. Lang. Writ. 49:100731. doi: 10.1016/j.jslw.2020.100731

[ref34] Jafarigohar M. Mortazavi M. (2013). The effects of different types of reflective journal writing on learners’ self-regulated learning. Iran. J. Appl. Linguist. 16, 59–78.

[ref35] Kellogg R. T. (1996). “A model of working memory in writing,” in The science of writing: Theories, methods, individual differences and applications. eds. Levy C. M. Ransdell S. (Hillsdale, NJ: Lawrence Erlbaum Associates), 57–71.

[ref36] Kormos J. (2012). The role of individual differences in L2 writing. J. Second. Lang. Writ. 21, 390–403. doi: 10.1016/j.jslw.2012.09.003

[ref37] Lee G. Schallert D. L. (2008). Meeting in the margins: effects of the teacher–student relationship on revision processes of EFL college students taking a composition course. J. Second. Lang. Writ. 17, 165–182. doi: 10.1016/j.jslw.2007.11.002

[ref38] Li Y. Flowerdew J. (2020). Teaching English for research publication purposes (ERPP): a review of language teachers’ pedagogical initiatives. Engl. Specif. Purp. 59, 29–41. doi: 10.1016/j.esp.2020.03.002

[ref39] Lillis T. Curry M. J. (2010). Academic Writing in a Global Context: The Politics and Practice of Publishing in English. London: Routledge.

[ref40] Locke E. A. Shaw K. N. Saari L. M. Latham G. P. (1981). Goal setting and task performance: 1969–1980. Psychol. Bull. 90, 125–152. doi: 10.1037/0033-2909.90.1.125

[ref41] Lv X. Ren W. Xie Y. (2021). The effects of online feedback on ESL/EFL writing: a meta-analysis. Asia Pac. Educ. Res. 30, 643–653. doi: 10.1007/s40299-021-00594-6

[ref42] Mallahi O. (2020). Examining the extent of self-regulatory strategy use and writing competence of EFL learners. Appl. Linguist. Res. J. 4, 13–23. doi: 10.14744/alrj.2020.70883

[ref43] Manchón R. M. (2011). Learning-to-write and writing-to-learn in an additional language. Amsterdam: John Benjamins.

[ref44] Mbato C. L. Cendra A. (2019). EFL undergraduate students’ self-regulation in thesis writing: help-seeking and motivation-regulation. JELE 5, 67–82. doi: 10.26486/jele.v5i1.949

[ref45] Moon J. A. (2006). Learning Journals: A Handbook for Reflective Practice and Professional Development 2nd *edn*. London: Routledge.

[ref46] Mu C. Carrington S. (2007). An investigation of three Chinese students’ English writing strategies. TESL-EJ 11, 1–23.

[ref47] Murray D. (1972). Teach writing as a process not product. Leaflet 71, 11–14.

[ref48] Oxford R. L. (2017). Teaching and Researching Language Learning Strategies: Self-regulation in Context. London: Routledge.

[ref49] Pallant J. (2020). SPSS Survival Manual: A Step by Step Guide to Data Analysis Using IBM SPSS 7th *edn*. London: Routledge.

[ref50] Pintrich P. R. (2000). “The role of goal orientation in self-regulated learning,” in Handbook of Self-regulation. eds. Boekaerts M. Pintrich P. R. Zeidner M. (Cambridge, MA: Academic Press), 452–502.

[ref51] Pintrich P. R. (2004). A conceptual framework for assessing motivation and self-regulated learning in college students. Educ. Psychol. Rev. 16, 385–407. doi: 10.1007/s10648-004-0006-x

[ref52] Rassaei E. (2015). Journal writing as a means of enhancing EFL learners’ awareness and effectiveness of recasts. Linguist. Educ. 32, 118–130. doi: 10.1016/j.linged.2015.10.002

[ref53] Saqr M. Peeters W. Viberg O. (2021). The relational, co-temporal, contemporaneous, and longitudinal dynamics of self-regulation for academic writing. Res. Pract. Technol. Enhanc. Learn. 16:Article 29. doi: 10.1186/s41039-021-00175-7

[ref54] Sasaki M. Mizumoto A. Murakami A. (2018). Developmental trajectories in l2 writing strategy use: a self-regulation perspective. Mod. Lang. J. 102, 292–309. doi: 10.1111/modl.12469

[ref55] Schunk D. H. (1990). Goal setting and self-efficacy during self-regulated learning. Educ. Psychol. 25, 71–86. doi: 10.1207/s15326985ep2501_6

[ref56] Schunk D. Ertmer P. A. (2000). “Self-regulation and academic learning: self-efficacy enhancing interventions,” in Handbook of Self-regulation. eds. Boekaerts M. Pintrich P. R. Zeidner M. (Cambridge, MA: Academic Press), 631–649.

[ref57] Sethuraman M. Radhakrishnan G. (2020). Promoting cognitive strategies in second language writing. Eurasian J. Educ. Res. 20, 1–17. doi: 10.14689/ejer.2020.88.5

[ref58] Shen B. Bai B. (2022). Chinese university students’ self-regulated writing strategy use and EFL writing performance: Influences of self-efficacy, gender, and major. Appl. Linguist. Rev. doi: 10.1515/applirev-2020-0103

[ref59] Sudirman A. Gemilang A. V. Kristanto T. M. A. (2021). The power of reflective journal writing for university students from the EFL perspective. Stud. Eng. Lang. Educ. 8, 1061–1079. doi: 10.24815/siele.v8i3.19105

[ref60] Sun Z. Lin C.-H. Lv K. Song J. (2021). Knowledge-construction behaviors in a mobile learning environment: a lag-sequential analysis of group differences. Educ. Technol. Res. Dev. 69, 533–551. doi: 10.1007/s11423-021-09938-x

[ref61] Sun T. Wang C. (2020). College students’ writing self-efficacy and writing self-regulated learning strategies in learning English as a foreign language. System 90:102221. doi: 10.1016/j.system.2020.102221

[ref62] Swales J. M. (2004). Research Genres: Explorations and Applications. Cambridge: Cambridge University Press.

[ref63] Takeuchi O. (2019). “Language learning strategies: insights from the past and directions for the future,” in Second Handbook of English Language Teaching. ed. Gao X. (Berlin: Springer), 683–702.

[ref64] Tavakol M. Dennick R. (2011). Making sense of Cronbach's alpha. Int. J. Med. Educ. 2, 53–55. doi: 10.5116/ijme.4dfb.8dfd, PMID: 28029643 PMC4205511

[ref65] Teng L. S. (2022). Explicit strategy-based instruction in L2 writing contexts: a perspective of self-regulated learning and formative assessment. Assess. Writ. 53:100645. doi: 10.1016/j.asw.2022.100645

[ref66] Teng L. S. Yuan R. E. Sun P. P. (2020). A mixed-methods approach to investigating motivational regulation strategies and writing proficiency in English as a foreign language contexts. System 88:102182. doi: 10.1016/j.system.2019.102182

[ref67] Teng L. S. Zhang L. J. (2016a). A questionnaire-based validation of multidimensional models of self-regulated learning strategies. Mod. Lang. J. 100, 674–701. doi: 10.1111/modl.12339

[ref68] Teng L. S. Zhang L. J. (2016b). Fostering strategic learning: the development and validation of the writing strategies for motivational regulation questionnaire (WSMRQ). Asia Pac. Educ. Res. 25, 123–134. doi: 10.1007/s40299-015-0243-4

[ref69] Teng L. S. Zhang L. J. (2018). Effects of motivational regulation strategies on writing performance: a mediation model of self-regulated learning of writing in English as a second/foreign language. Metacogn. Learn. 13, 213–240. doi: 10.1007/s11409-017-9171-4

[ref70] Thomas N. Bowen N. E. J. A. Rose H. (2021). A diachronic analysis of explicit definitions and implicit conceptualizations of language learning strategies. System 103:102619. doi: 10.1016/j.system.2021.102619

[ref71] Thomas N. Rose H. L. (2019). Do language learning strategies need to be self-directed? Disentangling strategies from self-regulated learning. TESOL Q. 53, 248–257. doi: 10.1002/tesq.473

[ref72] Thorpe K. (2004). Reflective learning journals: from concept to practice. Reflective Pract. 5, 327–343. doi: 10.1080/1462394042000270655

[ref73] Tseng W.-T. Dörnyei Z. Schmitt N. (2006). A new approach to assessing strategic learning: the case of self-regulation in vocabulary acquisition. Appl. Linguist. 27, 78–102. doi: 10.1093/applin/ami046

[ref74] Wenden A. (1991). Learner Strategies for Learner Autonomy. Hoboken, NJ: Prentice Hall.

[ref75] Wilby J. (2020). Motivation, self-regulation, and writing achievement on a university foundation programme: a programme evaluation study. Lang. Teach. Res. 26, 1010–1033. doi: 10.1177/1362168820917323

[ref76] Winne P. H. Hadwin A. E. (1998). “Studying as self-regulated learning,” in Metacognition in Educational Theory and Practice. eds. Hacker D. Dunlosky J. Graesser A. (Hillsdale, NJ: Lawrence Erlbaum Associates), 277–304.

[ref77] Wu C. P. Lin H. J. (2015). Examining the effects of conferencing and reflection paper in an EFL writing class. Int. J. Eng. Educ. 4, 289–298.

[ref78] Xu J. (2021). Chinese university students’ L2 writing feedback orientation and self-regulated learning writing strategies in online teaching during COVID−19. Asia Pac. Educ. Res. 30, 563–574. doi: 10.1007/s40299-021-00586-6

[ref79] Yabukoshi T. (2020). Self-regulated learning processes outside the classroom: insights from a case study of Japanese EFL students. J. Asia TEFL 17, 758–777. doi: 10.18823/asiatefl.2020.17.3.1.758

[ref80] Zhang L. J. (2010). A dynamic metacognitive systems account of Chinese university students’ knowledge about EFL reading. TESOL Q. 44, 320–353. doi: 10.5054/tq.2010.223352

[ref81] Zhang Y. Guo H. (2012). A study of English writing and domain-specific motivation and self-efficacy of Chinese EFL learners. Pan-Pacific Assoc. Appl. Linguist. 16, 101–121.

[ref82] Zhang R. Zou D. (2022a). Types, purposes, and effectiveness of state-of-the-art technologies for second and foreign language learning. Comput. Assist. Lang. Learn. 35, 696–742. doi: 10.1080/09588221.2020.1744666

[ref83] Zhang R. Zou D. (2022b). Self-regulated second language learning: a review of types and benefits of strategies, modes of teacher support, and pedagogical implications. Comput. Assist. Lang. Learn., 1–38. doi: 10.1080/09588221.2022.2055081

[ref84] Zimmerman B. J. (2000). “Attaining self-regulation: a social cognitive perspective,” in Handbook of Self-regulation. eds. Boekaerts M. Pintrich P. R. Zeidner M. (Cambridge, MA: Academic Press), 13–39.

[ref85] Zimmerman B. J. (2008). Investigating self-regulation and motivation: historical background, methodological developments, and future prospects. Am. Educ. Res. J. 45, 166–183. doi: 10.3102/0002831207312909

[ref86] Zimmerman B. Kitsantas A. (2002). Acquiring writing revision and self-regulatory skill through observation and emulation. J. Educ. Psychol. 94, 660–668. doi: 10.1037/0022-0663.94.4.660

[ref87] Zimmerman B. J. Risemberg R. (1997). Becoming a self-regulated writer: a social cognitive perspective. Contemp. Educ. Psychol. 22, 73–101. doi: 10.1006/ceps.1997.0919

[ref88] Zimmerman B. J. Schunk D. H. (2008). “Motivation: an essential dimension of self-regulated learning” in Motivation and Self-regulated Learning: Theory, Research, and Applications (Hillsdale, NJ: Lawrence Erlbaum Associates), 1–30.

[ref89] Zimmerman B. J. Schunk D. H. (2011). “Self-regulated learning and performance: an introduction and an overview,” in Handbook of Self-regulation of Learning and Performance (London: Routledge), 1–12.

