# Supplementary material for: On a path to becoming more self-regulated: Reflective journals’ impact on Chinese English as a foreign language students’ self-regulated writing strategy use
Source: Front Psychol. 2022 Nov 16;13:1042031. doi: 10.3389/fpsyg.2022.1042031 (PMC9710538; doi:10.3389/fpsyg.2022.1042031)
Supplement: Supplementary file 1 [file Table_1.docx]

**Appendix I. Questionnaire items on self-regulated writing strategy use**

| **Category** | **Subcategory** | **Items** |
| --- | --- | --- |
| Cognition | Text Processing (TP) | 1. When revising, I check grammar mistakes. 2. When revising, I check spelling and punctuation. 3. When revising, I check the structure for logical coherence. 4. When revising, I check the cohesiveness or connection among sentences. 5. When revising, I check whether the topic and the content have been clearly expressed. |
| Metacognition | Idea Planning (IP) | 1. I read related articles to help me plan. 2. I use the internet to search for related information to help me plan. 3. I think about the core elements of a good composition to help me plan. |
|  | Goal-oriented Monitoring (GM) | 1. When I learn English writing, I set up goals for myself in order to direct my learning activities. 2. I check my English learning progress to make sure I achieve my goal. 3. I evaluate my mastery of the content in writing courses. 4. I monitor my learning process in writing courses. 5. When I am writing, I tell myself to stick to my plan. 6. I set up a learning goal to improve my writing. |
| Social behavior | Peer Learning (PL) | 1. In writing courses, I brainstorm with peers to help me write. 2. I discuss with my peers or teachers to have more ideas to write. 3. I work with other students in writing courses. |
|  | Feedback Handling (FH) | 1. I am open to peers’ feedback on my writing. 2. I am open to teachers’ feedback on my writing. 3. I try to improve my English writing based on peers’ feedback. 4. I try to improve my English writing based on teachers’ feedback. |
| Motivational regulation | Interest enhancement (IE) | 1. I look for ways to bring more fun to the learning of writing. 2. I choose interesting topics to practice writing in English. 3. I connect the writing task with my real life to intrigue me. 4. I try to connect the writing task with my personal interest. |
|  | Mastery self-talk (MS) | 1. I persuade myself to work hard in writing courses to improve my writing skills and knowledge. 2. I persuade myself to keep on learning in writing courses to find out how much I can learn. 3. I persuade myself to work hard in writing courses to learn as much as possible. |
|  | Emotional Control (EC) | 1. I tell myself not to worry when answering questions in writing courses. 2. I tell myself to keep on completing a writing task when I want to give it up in writing courses. 3. I find ways to regulate my mood when I want to give up a writing task in writing courses. |
|  | Performance self-talk (PS) | 1. I tell myself that I need to do better than others in writing courses. 2. I tell myself that it is important to practice writing in English to outperform other peers. 3. I tell myself that it is important to get good grades in writing courses. 4. I tell myself that it is important to practice writing to get good grades in writing courses. |
