# Supplementary material for: On a path to becoming more self-regulated: Reflective journals’ impact on Chinese English as a foreign language students’ self-regulated writing strategy use
Source: Front Psychol. 2022 Nov 16;13:1042031. doi: 10.3389/fpsyg.2022.1042031 (PMC9710538; doi:10.3389/fpsyg.2022.1042031)
Supplement: Supplementary file 2 [file Table_2.docx]

**Appendix II. Guiding questions for reflective journals**

| **Reflective journal** | **Guiding questions** |
| --- | --- |
| The first reflective journal | 1. What are the differences between your first version and your current version of the assignment and how did your psychological state change in this process?  2. What are the strengths and weaknesses of your writing?  3. Which aspects require extra attention or practice in your future writing? |
| The second reflective journal | 1. What are the differences between your first version and your current version of the assignment and how did your psychological state change in this process?  2. What are the strengths and weaknesses of your writing?  3. Which aspects require extra attention or practice in your future writing? |
| The third reflective journal | 1. What are the differences between your first version and your final version of the assignment and how did your psychological state change in this process?  2. What changes did the repeated procedure of "writing-revising-writing-revising" bring to your writing and psychological state throughout the semester?  3. What changes did the peer review bring to your writing and psychological state throughout the semester?  4. What changes did writing reflective journals bring to your writing and psychological state throughout the semester? |
