# Supplementary material for: On a path to becoming more self-regulated: Reflective journals’ impact on Chinese English as a foreign language students’ self-regulated writing strategy use
Source: Front Psychol. 2022 Nov 16;13:1042031. doi: 10.3389/fpsyg.2022.1042031 (PMC9710538; doi:10.3389/fpsyg.2022.1042031)
Supplement: Supplementary file 3 [file Table_3.docx]

**Appendix III. Coding scheme for reflective journals**

| **Category** | **Subcategory** | **Definition** |
| --- | --- | --- |
| Metacognitive strategies | Self-monitoring and evaluation | Tracking and recording learning events and results; Reflecting on the learning process, comparing learner performance with goals and assessing learning quality |
|  | Idea planning | Arrange the content of writing in various ways |
|  | Goal-setting | Setting goals and sub-goals of learning |
| Cognitive strategies | Record reviewing | Recalling learning tasks and rereading learning materials |
|  | Perspective change | Think from the perspective of others (e.g. readers, teachers, etc.) |
|  | Contribution making | Making productive use of target knowledge and skills |
|  | Elaboration | Making the newly learned knowledge more meaningful and comprehensible by integrating it into prior knowledge |
|  | Text processing | Using linguistic knowledge (e.g. grammar, spelling, punctuation, clear expression, etc.) to revise or improve writing texts |
|  | Visualization | Organization of learning materials by making charts, diagrams, or tables |
| Motivational strategies | Emotional control | Addressing negative emotions, prompting motivation, enhancing learning awareness, and reducing stress during learning |
|  | Effort regulation | Improving and maintaining learning persistence when confronted with academic challenges |
|  | Self-consequence | Expecting or arranging rewards or punishment depending on the academic performance |
|  | Interest enhancement | Connecting personal interest with writing tasks to bring more fun to them |
|  | Performance self-talk | Talking to oneself of the need to do better than others and get good grades |
|  | Mastery self-talk | Persuading oneself to keep on working hard to know how much can be learnt and to improve writing skills and knowledge as much as possible |
| Behavioral strategies | Peer learning | Collaborating and communicating with other learners in academic projects |
|  | Seeking help | Soliciting instructional support from experts and other learners |
|  | Feedback handling | Responding to the instructional support offered by experts and other learners |
|  | Resource management | Seeking, identifying, and applying useful learning materials |
