# Supplementary material for: On a path to becoming more self-regulated: Reflective journals’ impact on Chinese English as a foreign language students’ self-regulated writing strategy use
Source: Front Psychol. 2022 Nov 16;13:1042031. doi: 10.3389/fpsyg.2022.1042031 (PMC9710538; doi:10.3389/fpsyg.2022.1042031)
Supplement: Supplementary file 4 [file Table_4.docx]

**Appendix IV. Coding scheme for students’ perceptions on the use of reflective journals**

| **Perception** | **Theme** | **Category** | **Quote** |
| --- | --- | --- | --- |
| Positive | Self-monitoring and evaluation | Summary | *“Reflective writing helps me summarize what I have learnt in the process of writing and revising, which would benefit my future writing.”* (Participant 24)  *“Reflective writing is not just to reflect, it's more to summarize the good things or the problems and mistakes during my writing process.”* (Participant 33) |
|  |  | Review | *“Reflective writing enables me to look back on what I have learnt and record my personal growth in a timely manner.”* (Participant 8)  *“Writing reflection helps me to reorganize as well as monitor the progress that I have made before the final submission of a writing assignment.”* (Participant 23) |
|  |  | Self-talk | *“Reflective writing is like a dialogue with myself.”* (Participant 30)  *“To me, Reflection is more like a tree hole, where I can put all my jokes and complaints about my writing.”* (Participant 24) |
|  |  | Self-recognition | *“Reflective writing gives me a chance to evaluate strengths and weaknesses in writing.”* (Participant 31)  *“Reflective writing is an opportunity for me to take myself to one side and examine myself.”* (Participant 17) |
|  |  | Reminder | *“Reflective writing reminds me to focus on my problems in writing and push me to think how I can do better next time.”* (Participant 16)  *“For me, reflection serves as a reminder.”* (Participant 11) |
|  |  | Record | *“In reflective writing, I would write down my thoughts before, during, and after writing my essays.”* (Participant 20)  *“I will record the important thoughts in my reflection.”* (Participant 20) |
|  | Goal-setting and future idea planning | Improvement | *“I always review my reflective writings before starting to write in order to make improvement and avoid problems in the past.”* (Participant 16)  *“In the next stage of writing, I will pay more attention and try to avoid the mistakes I made in the past.”* (Participant 34) |
|  |  | Inspiration | *“I am greatly inspired by reflective writing, from which I learn techniques and gain motivation for my future writings.”* (Participant 25)  *“Reflective writing gives me a lot of new inspirations in the process of writing (for example, I made progress in modifying the outline structure).”* (Participant 5) |
|  |  | Envisioning | *“Through reflective writing, I envision future studies in writing.”* (Participant 4)  *“I can find out my shortcomings and progress at each stage and come up with goals for the next stage through writing reflections.”* (Participant 26) |
|  | Motivation regulation | Accomplishment and satisfaction | *“Reflective writing helps me polish my essays, which gives me a sense of accomplishment.”* (Participant 20)  *“The process of writing reflection is a process that helps me from self-doubt to self-acceptance.”* (Participant 32) |
|  |  | Persistence | *“It turned out that so many boundaries of ‘I can't’ were actually set by myself and I can constantly break through these boundaries ...”* (Participant 31)  *“After the reflective writing, I realized that a lot of problems are not as difficult as I thought before, and everything can be solved once I find the root cause.”* (Participant 36) |
|  | Text-processing | Writing techniques | *“Through reflective writing, I pay close attention to my language, logic, and supporting facts. My writing greatly improves after my reflection.”* (Participant 27)  *“The first major contribution of reflective writing for students as writers is that such work allows us to examine tacit understandings to see where and how those might be elaborated for the complex or uncertain rhetorical contexts they write within.”* (Participant 23) |
|  | Social behavior | Instructor connection | *“Reflective writing enables me to create a close connection with my teacher. I have learnt a lot from her feedback to my reflective writing. She also encourages me a lot.”* (Participant 7)  *“I told my teacher about my anxiety and confusion through reflective writing and felt very lucky to get encouragement and suggestions from her.”* (Participant 25) |
| Negative |  |  | *“It is hard to find a quantitative standard, and I still cannot guarantee to get a higher score next time.”* (Participant 35)  *“Even if I am aware of my writing problems, I may make mistakes again in my next writing. Reflective writing is not an easy way to completely correct my mistakes.”* (Participant 33) |
