# Supplementary material for: On a path to becoming more self-regulated: Reflective journals’ impact on Chinese English as a foreign language students’ self-regulated writing strategy use
Source: Front Psychol. 2022 Nov 16;13:1042031. doi: 10.3389/fpsyg.2022.1042031 (PMC9710538; doi:10.3389/fpsyg.2022.1042031)
Supplement: Supplementary file 5 [file Table_5.docx]

**Appendix V. Samples of students’ reflective journals**

| **Reflective journal** | **Excerpts** |
| --- | --- |
| The first reflective journal | “*At first, I was confused and this confusion led to laziness and procrastination. Then I gradually wanted to choose a rather risky topic according to my own will. Next, I carefully, and even a little bit mechanically, combined materials into the first draft of the assignment. I found some major problems in this draft, however, on the night of the deadline, and decided to choose the road of no return: rewrite almost the whole article. After I figured out some proper directions, I had a better understanding of the requirements and methods introduced by the teacher. I realized that I was gradually taking the initiative to use these methods and techniques, and I was no longer overwhelmed by the rules as was the case at the beginning.*” (Participant 16) |
|  | “*I always think in a more rational way, so my writing is generally quite logical. I also enjoy reading my pieces of writing from the reader's perspective, which makes it easier for me to find my own writing problems. My shortcoming lies in the task of writing in English itself. Due to the lack of vocabulary and collocation knowledge as well as the experience of writing in English, I often get stuck when writing and have to use a dictionary or translation software for some words and sentences. I cannot express myself in English naturally and authentically, and examples of typical "Chinese English" often appear in my work.*” (Participant 34) |
|  | “*In my future writing, I need to further expand my vocabulary, focus on enriching the content, and write in a more academic way. I will continue to make up for my shortcomings in order to take a giant leap forward in terms of my writing level.*” (Participant 21) |
| The second reflective journal | “*When I wrote the outline this time, I did not save every draft after revision into a separate file. The reason was that I did much of the writing and revising work in my mind, and the outline was more like a kind of organization of various brainstorms. Through this outline, my strongest feeling was that along with my thinking about the outline, the idea of how to write the article had gradually become clear. I still could not plan the order of the subsequent sections well after I chose the topic and even after I wrote the introduction, but the process of building my outline step by step helped me clear my mind and provided me with great help to write the body of the article later.*” (Participant 9) |
|  | “*So far, my logical framework is quite clear. For outline, I think the more concise the better. More things need to be added to it, but there is no need for too much literature, or it will seem rather messy. I wrote the subheadings for each part in a “copy and paste” way because their logic was unified, and such a way of expression may help make reading smoother. There is a problem with concision: I am afraid that the content of my final paper will be too limited. In addition, I have not quite understood how to write the significance of the article.*” (Participant 28) |
|  | “*Writing this outline was a breakthrough in my own writing habits and made me realize its necessity, but such awareness still needs to be deepened in future practice. My outline still needs to be revised according to our teacher's comments. As I am going to write the first draft of my essay, I will focus on the development of the Gap and avoid discussing it too generally, and further adapt the examples to suit the theoretical framework to avoid rigid application. In the body part, after finding the theoretical framework, I still need to consider how to adapt the examples and the supporting theories according to each sub-argument. Ultimately, the conclusion section is still not clear enough, and it should be closely related to the perspectives chosen regarding the topic to draw relevant and meaningful conclusions. In addition, as our teacher mentioned in class, the concluding comments and suggestions that are not discussed in depth are no more than an act of painting the lily and can be deleted, and attention must be paid to this issue in my future academic writing.*” (Participant 26) |
| The third reflective journal | “*In draft 1 and the final essay, I did see a change in my logic. Our teacher and most of my classmates pointed out the issues that should be reconsidered. So I checked the structure, revised some paragraphs, and deleted the unneeded parts. I felt that my final essay did become more logical.*” (Participant 2) |
|  | “*At the psychological level, every time I revised, I kind of broke down, because I knew that I have to revise or even rewrite the article that I had struggled to finish. After the revision, however, I could see that my article was constantly being improved, and this process could bring me psychological satisfaction. The whole writing process made me feel that I was constantly improving my articles, just like an artist polishing his own work, and the immature articles kept getting better step by step. Although I still cannot write articles with perfect logic at this moment, it should be a goal for me to work on, and I will continue to use this way of writing essays to improve my writing ability.*” (Participant 30) |
|  | “*I could get many comments and suggestions through peer review by my classmates. They pointed out some problems in my essay and raised their questions as readers, and I would focus on these parts that caused confusion and try to clarify them in the process of revision. When I was reviewing other people's articles, I could learn from their strengths, find some shortcomings in their logical expressions and then reflect on my own writing. All in all, it was a two-way process in which both sides could learn and improve in terms of writing. Anyway, I was very impressed! I felt really grateful and touched to see how earnest my classmates were in their reviews, and we all gained a lot in this process. This kind of learning style of helping each other and making progress together is the most precious learning and interaction experience I gained this semester.*” (Participant 8) |
